# Evolution of Secondary Metabolites in *Eruca sativa* from the Microgreen to the Reproductive Stage: An Integrative Multi-Platform Metabolomics Approach

**DOI:** 10.3390/foods14234148

**Published:** 2025-12-03

**Authors:** Francesca Monzillo, Brigida Della Mura, Cristina Matarazzo, Maria Assunta Crescenzi, Sonia Piacente, Luigi d’Aquino, Rosaria Cozzolino, Paola Montoro

**Affiliations:** 1 Department of Pharmacy, University of Salerno, Via Giovanni Paolo II, 84084 Fisciano, Italy; fmonzillo@unisa.it (F.M.); mcrescenzi@unisa.it (M.A.C.); piacente@unisa.it (S.P.); 2 Ph.D. Program in Drug Discovery and Development, Department of Pharmacy, University of Salerno, Via Giovanni Paolo II, 84084 Fisciano, Italy; 3 Department of Science, University of Basilicata, Viale dell’Ateneo Lucano 10, 85100 Potenza, Italy; brigida.dellamura@unibas.it; 4 Department of Agriculture, Environmental and Food Sciences, University of Molise, 86100 Campobasso, Italy; 5 Institute of Food Science, National Council of Research (CNR), Via Roma 64, 83100 Avellino, Italy; rosaria.cozzolino@isa.cnr.it; 6 Italian National Agency for New Technologies Energy and Sustainable Economic Development (ENEA), Portici Research Centre, Piazzale E. Fermi 1, 80055 Portici, Italy; luigi.daquino@enea.it

**Keywords:** *Eruca sativa*, LC–HRMS, HS-SPME/GC–MS, precision agriculture

## Abstract

*Eruca sativa* Mill. (rocket; Fam. Brassicaceae) is widely appreciated for its peculiar flavour and beneficial effects on human health. Glucosinolates (GSLs) and their enzymatic hydrolysis products, isothiocyanates (ITCs), are considered to be responsible for health-promoting effects and for sensory relevance in rocket, respectively. This study aimed at evaluating and comparing the metabolite profiles of rocket leaves collected at different phenological stages, to investigate the content evolution during cultivation. To minimise metabolic variability induced by environmental factors, plants were cultivated in an innovative growing system equipped with precision lighting and ventilation. A multi-platform metabolomics approach combining liquid chromatography–high-resolution mass spectrometry (LC–HRMS) and headspace solid-phase microextraction coupled with gas chromatography–mass spectrometry (HS-SPME/GC–MS) was carried out for comprehensive coverage of non-volatile and volatile organic compounds (VOCs). To integrate data from both platforms, a multivariate data fusion strategy was used. Higher GSLs content was detected in the microgreens stage. In particular, glucoraphanin, glucoiberverin, glucoerucin, DMB-GLS, and 1,4-dimethoxyglucobrassicin were identified as biological markers of rocket microgreens. ITCs levels were found to increase in mature leaves. These findings suggest a dynamic modulation of secondary metabolism during the plant life cycle, possibly in response to different adaptation needs to environmental conditions. Our findings confirm the potential of microgreens as a functional food in promoting health and preventing chronic diseases and can also tailor rocket cultivation to maximise the production of beneficial metabolites and to improve selected sensorial features.

## 1. Introduction

The human population increase, urbanization spreading, decline of soil fertility, water scarcity, and climate change threaten food security at a global level. To face the reduced availability and fertility of arable land, various innovative agricultural systems to grow plants in unconventional places, i.e., locations where plant growing is not currently feasible, such as indoor environments, have been proposed [1]. Such cultivation systems are widely based on precision agriculture approaches and fine regulation of environmental factors, markedly light supply, has been reported to be highly effective in driving the secondary metabolism of plants [2]. Microgreens, i.e., vegetables harvested just after cotyledon expansion and emission of the first two true leaves, are well-suited for such cultivation systems, thanks to their reduced size and short cultivation period. The popularity of these vegetables is increasing due to their appealing sensory traits and high nutritional value. In fact, previous studies reported that microgreens contain higher levels of essential nutrients and bioactive compounds compared to mature plant tissues, although differences can be species- and environment-dependent [3].

Recent studies on Brassicaceae species have shown that the accumulation of secondary metabolites is critically influenced by both plant developmental stage and growing environment [4]. For instance, young leaves of *Brassica oleracea* var. *capitata* accumulate higher levels of indolic glucosinolates than mature leaves or stems [5], whereas aliphatic glucosinolates in *B. oleracea* var. *italica* (broccoli) decrease from seed to seedling stage, with indolic types peaking at the early seedling stage [6].

Environmental factors, including light intensity, nutrient availability and abiotic stresses such as salinity, further modulate the total content and specific profiles of flavonoids and glucosinolates [4]. Similar trends have been observed in other Brassicaceae species, suggesting that intrinsic developmental cues and external growing conditions both influence phytochemical composition. These findings emphasise the importance of considering plant phenology and cultivation environment when studying secondary metabolites in Brassicaceae. *Eruca sativa* Mill. (Fam. Brassicaceae), the so-called “arugula” or “rocket”, is widely cultivated for the distinctive pungent flavour of the edible green leaves [7,8]. Beyond culinary uses in salads, sauces, and pesto, *E. sativa* displays a long history in traditional medicine [9]. Rocket nutraceutical potential has recently met an increasing interest in the scientific community due to its high content of health-promoting phytochemicals, including vitamins, minerals, carotenoids, flavonoids, and glucosinolates (GSLs) which extend anti-inflammatory, diuretic, and digestive effects [8].

GSLs are sulfur- and nitrogen-containing secondary metabolites typical of Brassicaceae species. Although biologically inert in their intact form, they are hydrolysed upon tissue damage by the endogenous enzyme myrosinase, generating various bioactive compounds, including isothiocyanates (ITCs) [10,11]. ITCs have been widely studied for their sensory impact, since they contribute to the characteristic pungency of rocket leaves, as well as for their potential health benefits, including antioxidant and chemo-preventive activities [12].

The concentration and composition of GSLs and ITCs in *E. sativa* are influenced by several factors, such as developmental stage, environmental conditions, and cultivation practices [13]. Despite the relevance of these bioactive compounds, there is still limited knowledge about their dynamic distribution and transformation during the life cycle of *E. sativa*. Most studies focused on mature plants, while investigation on metabolites accumulation in different developmental stage has so far been underrated. Nevertheless, the knowledge about the accumulation of GSLs and ITCs during the plant evolution would be very interesting to optimise, by using cultivation approaches for boosting the content of selected phytochemicals. The aim of this study was to characterise and compare the bioactive metabolites composition of *E. sativa* at microgreen and fully expanded leaves stages, either before or during the transition of the plants from the vegetative to the reproductive stage. Special attention was given to the modulation of GSLs and ITCs during plant development. Extracts from leaves collected at different phenological stages were analysed using ultra-high-performance liquid chromatography coupled with high-resolution mass spectrometry (UHPLC-Q-Exactive-MS/MS) in negative ion mode. Volatile organic compounds (VOCs) profiling was also performed on fresh tissues via headspace solid-phase microextraction combined with gas chromatography–mass spectrometry (HS-SPME/GC–MS). A multivariate data analysis approach was used, to identify metabolic trends, putative discriminant markers, and stage-specific biochemical shifts.

## 2. Materials and Methods

### 2.1. Germplasm and Growing Conditions

**Figure 1:**
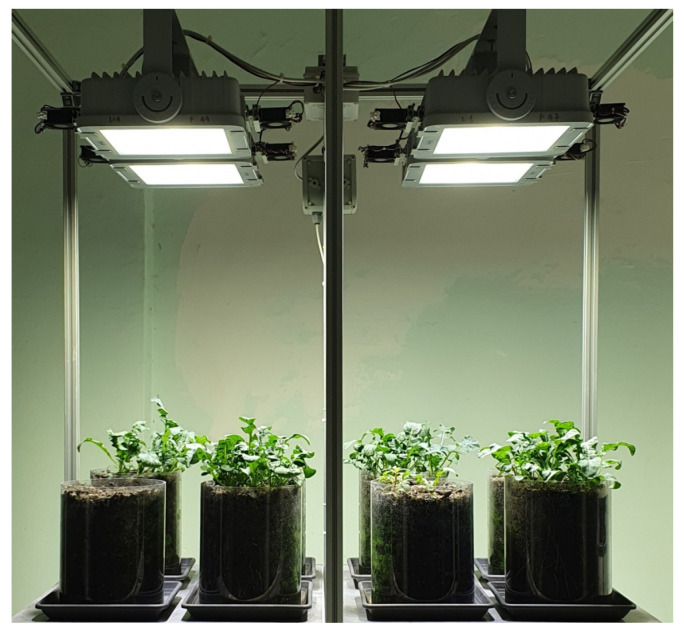
Ventilating lamp set-up for plant growing.

For plant growing, an innovative cultivation set-up based on conventional root substrate, precision lighting, and targeted ventilation, developed by the Italian National Agency for New Technologies Energy and Sustainable Economic Development (ENEA) named “Ventilated Lamp set-up”, was used (Figure 1). Briefly, the set-up was made up of polycarbonate cylindrical pots (about 19 cm wide and 25 cm deep) for substrate housing placed under precision LED lamps equipped with fans for an even and constant ventilation of the plants.

Before seeding, the pots were filled with a VigorPlant^®^ Completo^®^ (www.vigorplant.com) substrate (peat 63% + coconut granules 23% + pumice 14%, pH 6.5, electrical conductivity 0.35 dS/m, porosity 92% *v*/*v*), enriched with 0.2 g potassium phosphate monobasic, 0.2 g potassium phosphate dibasic, 0.2 g potassium sulphate, 0.2 g magnesium sulphate and 0.2 g iron sulphate.

Seeds of *E. sativa* were provided by La Semiorto Sementi S.r.l. (Sarno, Italy). Twelve seeds per pot were sown along three rows evenly spaced along the substrate surface and allowed to germinate in the dark.

After germination, a white light spectrum was chosen for plant lighting. Photosynthetic photon flux density was measured at about 5 cm from the substrate surface, i.e., at about 90 cm from the light source using a LI-190R Quantum Sensor and LI-1500 Light Sensor Logger (LI-COR Biosciences, Lincoln, NE, USA) and was set at 230 µmol·m^−2^·s^−1^ along the whole growing cycle.

**Figure 2:**
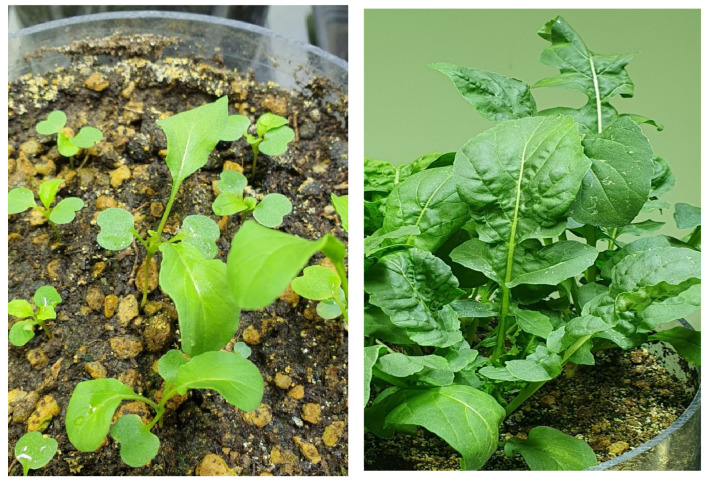
Plants M (**left**) and V (**right**) at the harvest moment.

In a first experiment aimed at growing plants to be harvested at the microgreen stage (plants M) and at the vegetative stage (plants V), photo- and thermo-period were 16 h/8 h light/dark and 26 °C/30 °C night/day, respectively, thus simulating South Italian summer cultivation conditions. The plants M and V were watered with 1.7 L water and 3.0–4.5 L per pot during the growing period, respectively. No fertigation was carried out for plants M, whereas plants V were fertilised once with Fertiactyl GZ^®^ (Timac Agro, Milano, Italy) 1%. The plants M were harvested 14 days after germination, when all the plants displayed two true leaves widely spread plus cotyledons (Figure 2 left), whereas plants V were harvested 35 days after germination, when all the plants showed well expanded leaves and none of them had started the inflorescence emission (Figure 2 right).

**Figure 3:**
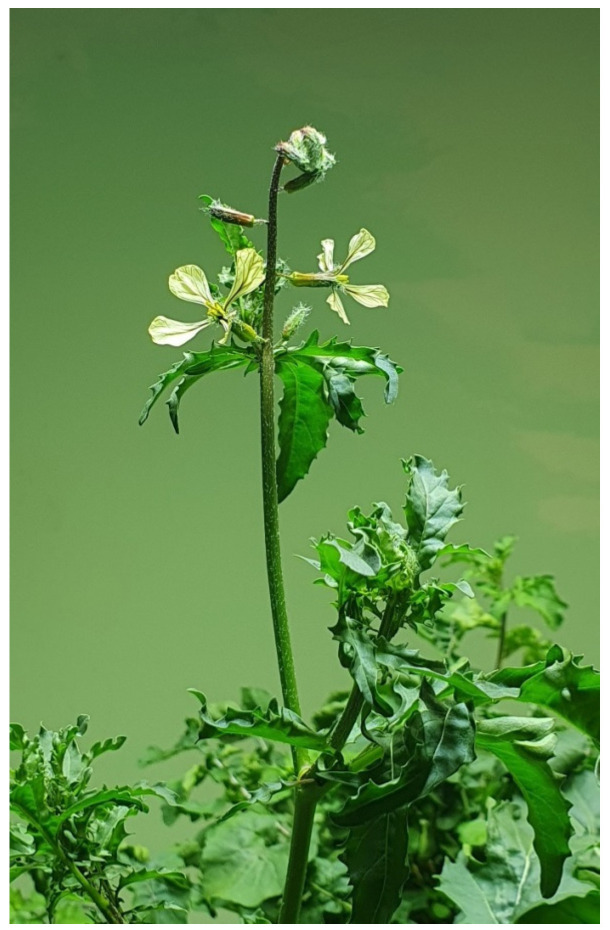
Plants R at the harvest moment.

In a second experiment aimed at growing plants to be harvested at the reproductive stage (plants R), the photo- and thermo-period were 12 h/12 h light/dark and 10 °C/16 °C night/day, respectively, thus simulating Italian winter-to-spring cultivation conditions, The plants were watered with 3.7–4.2 L water per pot and fertilised once with Fertiactyl GZ^®^ (Timac Agro, Milano, Italy) 2.5% and once with ammonium sulphate 2 g/L during the growing period. The plants were harvested 73 days after germination, when all the plants had emitted inflorescence axes and anthesis occurrence had already started (Figure 3).

### 2.2. Reagents and Solvents

Ethanol and water used for the extractions were purchased from VWR (Milan, Italy). Acetonitrile (ACN), formic acid, water, and methanol of LC–MS grade were purchased from Merck (Merck KGaA, Darmstadt, Germany).

### 2.3. Sample Preparation

After collection, fresh samples from plants M, V, and R were immediately pulverised in a mortar with liquid nitrogen and stored at −80 °C until further processing. An aliquot of 150 mg from each sample was extracted with 2 mL of an ethanol:water (7:3, *v*/*v*) solution using ultrasound-assisted extraction (UAE) for 15 min at room temperature, operating at 200 W of power with a frequency of 44 kHz. The mixture was then centrifuged at 2750 rpm for 5 min using a MiniSpin plus centrifuge (Eppendorf, Hamburg, Germany) to remove coarse residues. The resulting supernatant was dried under a gentle nitrogen stream and reconstituted in methanol:water (1:1, *v*/*v*; LC–MS grade) to a final concentration of 1 mg/mL.

### 2.4. UHPLC-Q-Exactive/MS and UHPLC-Q-Exactive/MS/MS Analysis

Aliquots of 10 µL of each hydroalcoholic extract (1 mg/mL) were analysed using a metabolite profiling approach based on an ultra-high-performance liquid chromatography (UHPLC) system coupled to a Q-Exactive high-resolution mass spectrometer (Thermo Fisher Scientific, Bremen, Germany) equipped with an electrospray ionization source operating in negative ion mode. The experiments were performed under the conditions described in a recent article by Crescenzi M.A. et al., 2023 [14]. Chromatographic separation was achieved on a Kinetex EVO C18 column (150 × 2.1 mm, 5 µm particle size; Phenomenex, Aschaffenburg, Germany).

The mobile phases used were water with 0.1% formic acid (A) and acetonitrile with 0.1% formic acid (B). An increasing linear gradient (*v*/*v*) of solvent B was applied at a flow rate of 0.200 mL/min as follows: 0–23 min, from 5 to 40%; 23–45 min, from 40 to 95%; then returning to 5% for 10 min. The ESI source parameters were as follows: spray voltage, 2.5 kV; capillary temperature, 300 °C; sheath gas (N_2_), 50 a.u.; auxiliary gas, 10 a.u.; sweep gas, 0 a.u.; probe heater temperature, 300 °C; and S-lens RF voltage, 50 a.u.

The MS spectra were acquired over a mass range of 150–1400 *m*/*z* with a resolution of 70,000. To obtain (HR)MS/MS spectra a data-dependent scan (ddMS2) experiment was performed where the five most intense precursor ions were selected for fragmentation in the LC–MS analysis using Collision-Induced Dissociation (CID) at 30%. Xcalibur™ software version 2.2 (Thermo Fisher Scientific, Build 2806, Bremen, Germany) was used for instrument control and data acquisition, while data analysis was combined with Compound Discoverer™ software version 3.3 SP3 (Thermo Fisher Scientific, Bremen, Germany).

### 2.5. Compound Discoverer Data Analysis

UHPLC-Q-Exactive-MS/MS data obtained in negative ion mode were processed using Compound Discoverer™ 3.3 SP3.

The Input Files node was used to import raw data files, including both samples and blanks. Files were categorised by type and experimental group to enable background subtraction and batch correction. To monitor system performance and maintain data quality, pooled QC samples (*n* = 3) were analysed throughout the sequence.

The Select Spectra node was configured to include only negative ion mode scans. MS^1^ and MS^2^ spectra were retained in the 150–1400 *m*/*z* range within the retention time window of 0–50 min. Retention times were aligned across all samples using the Align Retention Times node with the adaptive curve algorithm. A maximum RT shift of 0.5 min and a mass tolerance of 5 ppm were applied to ensure accurate peak matching.

Compound detection was performed with the Detect Compounds node using a mass tolerance of 5 ppm, minimum peak intensity of 100,000, at least four scans per peak, and a signal-to-noise threshold of 1.5. The adducts considered included [M − H]^−^, [M + FA − H]^−^ and [M − 2H]^2−^, appropriate for negative ion mode with formic acid in the mobile phase.

Detected compounds in each sample were grouped using the Group Compounds node with a mass tolerance of 5 ppm and a retention time tolerance of 0.2 min. The peak alignment option was disabled to preserve the retention time corrections applied in the preceding alignment node.

The Fill Gaps node was applied to recover missing features across samples, using a 5 ppm mass tolerance to ensure consistent compound detection in the data matrix.

The Mark Background node flagged features present in blanks to identify background signals, using a Sample/Blank ratio of 5, a Blank/Sample ratio of 0, and hiding background features to improve data quality.

Compound identification was enhanced by the Search mzCloud node, which matched MS/MS spectra against the mzCloud spectral library using the HighChem HighRes algorithm. Searches spanned all compound classes with precursor and fragment mass tolerances set at 10 ppm, without additional filtering to maximise identification coverage.

The Predict Compositions node was set with a mass tolerance of 2 ppm and maximum elemental counts as follows: C_90_, H_180_, N_5_, O_40_, P_5_, S_5_. These limits reflect the expected chemical space of plant metabolites.

For the Search ChemSpider node, databases selected to cover plant metabolomics included BioCyc, PlantCyc, Phenol-Explorer, FooDB, and MassBank.

The Apply mzLogic node integrated spectral matching, isotope patterns, and predicted compositions to refine compound identifications using a 10 ppm mass tolerance. Additional nodes—Apply Spectral Distance, Merge Features, and Assign Compound Annotation—were included.

In the Assign Compound Annotation node, five data sources were used: mzCloud Search, mzVault Search, MassList Search, Predicted Compositions, and ChemSpider Search.

The Search Mass Lists node utilised the Arita Lab 6549 Flavonoid Database.masslist with a 5 ppm mass tolerance. Finally, the Calculate Mass Defect node was added with default parameters.

### 2.6. GC-qMS Analysis

VOCs were extracted by HS-SPME and analysed by GC/MS as follows. In a 20 mL headspace glass vial with a screw-top PTFE septum (Supelco^®^, Bellefonte, PA, USA), 1 g of each different sample was weighted. Migration of VOCs to HS was prompted by putting the sample at 40 °C for 10 min in the dry block-heater of the GC instrument. VOCs adsorption was achieved by inserting a DVB/CAR/PDMS (50/30 µm) fiber into the vial for 20 min at 40 °C. For VOCs desorption, the fiber was mechanically introduced into a split-splitless injector, at 230 °C for 10 min, of an Agilent 7890A GC (7890A, Agilent Technologies, Santa Clara, CA, USA) hyphenated to a 5975A MS (5975A, Agilent Technologies, Santa Clara, CA, USA) and separated by using a capillary column HP-Innowax (30 m × 0.25 mm × 0.5 μm) (Supelco^®^, Bellefonte, PA, USA).

Oven temperature was first set at 50 °C for 2 min, then at 150 °C at 10 °C min^−1^ and 240 °C at 15 °C min^−1^. Helium at a flow rate of 1 mL min^−1^ served as the carrier gas. Ion source and quadrupole temperatures were set at 230 and 150 °C, respectively, while the MS operated at 70 eV. VOCs were identified or tentatively identified by comparing the mass spectra and the retention times using the available libraries (NIST, version 2005; Wiley, version 2007) and by matching their retention times with pure standards, when commercially available. Additionally, VOCs identification was also achieved by their retention indices (LRI) (as Linear Retention Index), measured by using a C_8_–C_22_ n-alkanes series. Samples were analysed in triplicate and results were reported as semi-quantitative data (Relative Peak Area, RPA%) respect to the peak area of 3-octanol (IS). The area of each VOC was calculated from the total ion current (TIC).

### 2.7. Multivariate Data Analysis

To investigate differences in the *E. sativa* leaf metabolome across the three phenological stages, multivariate statistical analyses (MVAs) were performed using both Principal Component Analysis (PCA) and Partial Least Squares Discriminant Analysis (PLS-DA).

LC-Q-Exactive/MS chromatograms acquired in negative ion mode were processed using the open-source software MZmine version 2.10 (http://mzmine.sourceforge.net/ (accessed on 25 May 2025)). Noise was removed from the LC–MS profiles, and signals with intensities below 5.0 × 10^6^ were excluded. The processed data were exported in tabular (.csv) format, generating a data matrix for subsequent analysis.

GC–MS data were processed following a pseudo-targeted approach. Peak areas corresponding to identified volatile compounds were used to construct the data matrix, which was also exported in tabular format. Prior to multivariate analysis, raw LC-ESI-MS and GC–MS data were log-transformed to reduce skewness and normalised using unit variance (UV) scaling to address heteroscedasticity and improve interpretability.

Multivariate analyses were conducted using SIMCA^®^-P software version 12.0 (Umetrics, Umeå, Sweden). PCA and PLS-DA were applied separately to the LC–MS and GC–MS datasets. Model validation was performed using cross-validation and permutation testing in line with recommended standardization practices to minimise overfitting and ensure robust statistical models.

For integrative analysis, a low-level data fusion approach was employed by combining the GC–MS and LC–MS datasets into a single matrix. Prior to fusion, GC–MS, and LC–MS variables (areas under the curve) were normalised using UV scaling to balance their relative contributions. PCA was then applied to the fused dataset in SIMCA^®^-P+ 12.0 to explore sample clustering based on overall metabolite composition. Finally, PLS-DA was applied to the fused dataset to identify potential chemical markers differentiating the developmental stages. Numerical scores were assigned to the three sample classes (M = −1; V = 0; R = +1) to enable regression-based classification.

Model performance was evaluated using cross-validation with R^2^ and Q^2^ metrics, and permutation testing. Variable Importance in Projection (VIP) scores were used to select the most discriminant metabolites.

The HS-SPME/GC–MS data were subjected to an univariate statistical analysis (Anova, *p* < 0.05), using MetaboAnalyst (Xia Lab, McGill University, Montreal, QC, Canada). Briefly, the raw data were treated by IS ratio correction, sample median and data scaling by autoscaling. The Anova was carried out to assess significant statistical differences among the VOCs’ profiles in each plant sample group (*p* < 0.05; Supplementary Table S1).

## 3. Results

### 3.1. UHPLC-Q-Exactive/MS and UHPLC-Q-Exactive/MS/MS Analysis

**Figure 4:**
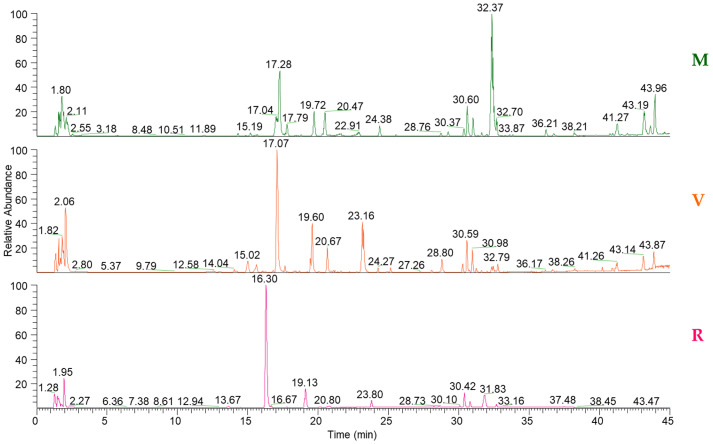
UHPLC-Q-Exactive-MS profiles in negative ion mode of plant extracts at the microgreen (M), vegetative (V), and reproductive (R) stages.

LC-ESI-Q-Exactive-MS and LC-ESI-Q-Exactive-MS/MS profiles of *E. sativa* hydroalcoholic extracts were previously analysed by Xcalibur™ 2.2 software to generate accurate masses for the preliminary identification of compounds with suitable confidence (ppm ≤ 5). High-resolution analysis data combined with fragmentation pattern analysis (MS/MS) and score-based matching were used to annotate the compounds by the Compound Discoverer tool linked to customised workflows, as described in the experimental section. Annotations were further supported by literature data, as reported in Table 1. Figure 4 shows the total ion current LC–MS chromatograms obtained from *E. sativa* extracts at the microgreen (M), vegetative (V), and reproductive (R) stages. Metabolomic profiling of these extracts led to the annotation of 52 compounds. All compounds were classified as level 2 identifications, according to the criteria of the Metabolomics Standards Initiative (MSI) [15].

Overall, the samples were rich in bioactive compounds, mainly glucosinolates (**6**, **10**, **12**, **16**, **27**, **31**, **32**, **35**, **36**, **40**, **46**), glycosylated flavonoids (**17**, **21**–**25**, **29**, **30**, **33**), glycolipids (**42**–**45**, **47**, **51**, **52**), hydroxycinnamic acids (**15**, **18**, **19**), and fatty acids (**34**, **38**, **49**).

Compound **13**, putatively identified as mono[(1-thioxo-4-penten-1-yl)azanyl] ester, is potentially derived from glucosinolate degradation and is characterised by a diagnostic fragment at *m*/*z* 95.9509, consistent with a sulfur-containing structure featuring thiol and/or isothiocyanate groups.

Quercetin and kaempferol derivatives (**24** and **29** for quercetin compounds **17**, **21**, **22**, **25**, **30,** and **33** for kaempferol) were confidently annotated based on LC–HRMS/MS data processed using Compound Discoverer™ software. The identification was supported by diagnostic fragmentation patterns obtained in negative ion mode, with aglycone nominal ions at *m*/*z* 301 and 285, respectively, resulting from the sequential neutral losses of sugar moieties (e.g., hexose, rhamnose, glucuronic acid) and acyl groups (e.g., sinapoyl, feruloyl). These fragmentation signatures are consistent with previously reported data and confirm the occurrence of highly glycosylated and acylated flavonol conjugates [16,17].

Compounds **34** and **38** were identified as oxylipins, i.e., hydroxylated fatty acids differing in degree of unsaturation and number of hydroxyl groups [12].

Compound **48**, characterised by the heteroatom composition NO_7_P, was annotated as l-PE 16:0 based on the observed neutral loss of Fatty_Acid_16:0.

Some compounds (**35**, **40**, **46**), not yet reported in the literature, were attributed as glucosinolates due to their distinctive isotope pattern—related to the presence of sulfur atoms in their structure—and the occurrence of specific fragmentation pathways ([SO_4_]^•−^ at nominal *m*/*z* 96 or the [HSO_4_]^−^ ion at nominal *m*/*z* 97 during MS^2^ analyses [18].

**Table 1:** Metabolite annotation in *E. sativa* extracts by UHPLC-Q-Exactive-MS/MS analysis in order of elution in extracts of *E. sativa* at microgreen (M), vegetative (V), and reproductive (R) stages.

N°	Rt (min)	[M − H]^−^ (*m*/*z*)	Molecular Formula	RMS Error (ppm)	MS/MS (*m*/*z*)	Compound	M	V	R	Reference
**1**	1.57	195.0502	C_6_H_12_O_7_	0.9	75.0073/129.0180	Gluconic acid	✓	✓	✓	[19]
**2**	1.66	341.1088	C_12_H_22_O_11_	2.9	179.0549/161.0444/143.0337	Sucrose	✓	✓	✓	[20]
**3**	1.82	333.0594	C_9_H_19_O_11_P	−3.2	152.9950/241.0120/259.0239/181.0056/78.9576	Phosphatidyl-myoinositol	✓	✓	✓	[21]
**4**	2.11	191.0187	C_6_H_8_O_7_	0.3	111.0074/87.0073/129.0181	Isocitric acid	✓	✓	✓	[22]
**5**	2.36	323.0289	C_9_H_13_O_9_N_2_P	2.5	211.0010/78.9579/111.0189/96.9685	5′-UMP	ND	ND	✓	[23]
**6**	2.57	436.0409	C_12_H_23_O_10_NS_3_	0.7	96.9587/95.9509/178.0170/372.0423/74.9895/79.9558/259.0118	Glucoraphanin	✓	ND	✓	[14]
**7**	3.19	565.0479	C_15_H_24_O_17_N_2_P_2_	2.2	323.0276/78.9577/96.9681/241.0116/158.9246/211.0012/ 272.9576/305.0149	Uridine-5′-diphospho glucose	✓	✓	✓	[24]
**8**	3.56	344.0402	C_10_H_12_O_7_N_5_P	3.2	150.0409/133.0148	Cyclic GMP	✓	✓	ND	[25]
**9**	4.66	315.0724	C_13_H_16_O_9_	4.0	152.0102/108.0202	Genistic acid glucoside	✓	✓	ND	[14]
**10**	10.51	406.0307	C_11_H_21_O_9_NS_3_	2.9	96.9587/95.9508/74.9896/79.9560/259.0119/114.0547	Glucoiberverin	✓	ND	✓	[14]
**11**	11.76	461.1311	C_19_H_26_O_13_	3.4	163.0392/167.0338	Saccharumoside C	✓	✓	ND	[26]
**12**	11.89	711.0999	C_21_H_36_O_15_N_4_S_4_	3.1	96.9587/306.0763/143.0451/179.0452/456.1617	Glutathione disulfanyl butyl-GLS	✓	ND	✓	[27]
**13**	12.20	209.9895	C_5_H_9_O_4_NS_2_	2.8	95.9509	Sulfuric acid, mono[(1-thioxo-4-penten-1-yl)azanyl] ester	✓	✓	ND	[14]
**14**	12.92	395.0291	C_13_H_16_O_12_S	−3.2	153.0181/241.0020	Protocatechuic acid-*O*-sulfate-*O*-glucoside	✓	✓	ND	[28]
**15**	13.71	325.0930	C_15_H_18_O_8_	3.4	145.0282/163.0391	*p*-Coumaric acid 4-*O*-glucoside	✓	ND	ND	[29]
**16**	14.15	420.0465	C_12_H_23_O_9_NS_3_	2.0	96.9587/74.9896/95.9508/128.0341/79.9560/259.0119	Glucoerucin	✓	ND	ND	[14]
**17**	14.32	817.2048 [(M + FA) − H]^−^	C_33_H_40_O_21_	1.8	609.1461/447.0930/285.0402	Kaempferol 3-diglucoside 7-glucoside	✓	✓	✓	[30]
**18**	14.91	355.1037	C_16_H_20_O_9_	3.8	175.0391/193.0505	Ferulic acid 4-*O*-glucoside	✓	✓	✓	[31]
**19**	15.19	385.1143	C_17_H_22_O_10_	2.1	205.0499/190.0263	Sinapic acid glucoside	✓	✓	ND	[14]
**20**	16.10	371.0986	C_16_H_20_O_10_	3.4	121.0282/249.0620/113.0230	Deacetylasperuloside	✓	ND	ND	[32]
**21**	16.28	771.2015	C_33_H_40_O_21_	4.0	285.0403	Kaempferol 3-triglucoside	ND	✓	✓	[33,34]
**22**	17.27	609.1442	C_27_H_30_O_16_	−1.2	285.0404/283.0243/476.0854	Kaempferol-3,4′-diglucoside	✓	✓	✓	[30]
**23**	17.58	639.1570	C_28_H_32_O_17_	1.4	313.0352/476.0952/477.1036/285.0403	Isorhamnetin-3,4′-diglucoside	✓	ND	ND	[30]
**24**	17.70	993.2512	C_44_H_50_O_26_	0.5	301.0349/463.0876	Quercetin-3,4′-diglucoside 3′-(6-sinapoylglucoside)	✓	ND	ND	[14]
**25**	17.79	977.2557	C_44_H_50_O_25_	0.2	285.0399/653.1470/447.0950/429.0864	Kaempferol 3-*O*-sinapoylsophoroside 7-*O*-glucoside	✓	✓	✓	[35]
**26**	18.41	521.2034	C_26_H_34_O_11_	2.1	341.1386	Lariciresinol-4′-glucoside	ND	✓	✓	[36]
**27**	18.8	477.0646	C_17_H_22_O_10_N_2_S_2_	2.8	96.9587/95.9509/74.9896/79.9559/259.0110	Neoglucobrassicin	✓	✓	✓	[14]
**28**	19.22	187.0971	C_9_H_16_O_4_	0.1	125.0959/123.0800/143.1068	Azelaic acid	ND	✓	ND	[19]
**29**	20.31	831.2001	C_38_H_40_O_21_	2.7	301.0349/463.0883	Quercetin 3-(6‴-sinapylhexoside-hexoside	✓	✓	ND	[37]
**30**	20.67	815.2028	C_38_H_40_O_20_	−0.1	285.0403/447.0930	Kaempferol 3-(2-sinapoylglucoside) 4′-glucoside	ND	✓	ND	[30]
**31**	20.67	386.0406	C_12_H_21_O_9_NS_2_	2.1	74.98974/96.9587/95.9508/79.9560/259.0119	Glucobrassicanapin	ND	✓	ND	[38]
**32**	20.72	405.0229 [M − 2H]^2−^	C_22_H_40_O_18_N_2_S_6_	−1.5	96.9587/95.9509/74.9896/259.0131	DMB-GLS	✓	ND	✓	[14]
**33**	20.85	785.1945	C_37_H_38_O_19_	2.7	285.0401	Kaempferol 3-(feruloyldiglucoside)	✓	✓	ND	[39]
**34**	24.39	327.2176	C_18_H_32_O_5_	2.9	211.1331/229.1440/171.1019	Trihydroxy octadecadienoic acid	✓	✓	✓	[14]
**35**	22.49	504.1045	C_17_H_31_O_10_NS_3_	2.6	96.9590/74.9898	Unknown	ND	ND	✓	-
**36**	22.78	507.0754	C_18_H_24_O_11_N_2_S_2_	2.3	96.9588	1,4-Dimethoxyglucobrassicin	ND	✓	✓	[40]
**37**	25.35	242.1760	C_13_H_25_O_3_N	3.2	225.1494/181.1587	*N*-Octanoyl-L-valine	✓	✓	ND	[41]
**38**	25.56	329.2335	C_18_H_34_O_59_	1.2	211.1333/229.1438	Trihydroxy-octadecanoic acid	✓	✓	ND	[14]
**39**	27.04	253.1446	C_14_H_22_O_4_	4.7	209.1540	*β*-caryophyllinic acid	ND	✓	ND	[42]
**40**	28.54	713.0869	-	-	96.9587/95.9509/456.8649/373.2574	Unknown	✓	ND	ND	-
**41**	29.26	293.1761	C_17_H_26_O_4_	4.2	211.1540/236.1047/96.9586	Gingerol	✓	✓	ND	[14]
**42**	30.37	531.2808 [(M + FA) − H]^−^	C_25_H_42_O_9_	1.5	249.1855	MGMG 16:3	✓	✓	ND	[14]
**43**	30.61	721.3648 [(M + FA) − H]^−^	C_33_H_56_O_14_	1.3	277.2169/397.1354/89.0230/59.0125/101.0230/71.0125	DGMG 18:3	✓	✓	✓	[14]
**44**	31.65	723.3821 [(M + FA) − H]^−^	C_33_H_58_O_14_	3.9	279.2327	Gingerglycolipid B	✓	✓	✓	[43]
**45**	32.25	699.3821 [(M + FA) − H]^−^	C_31_H_58_O_14_	2.7	255.2331/89.0232/397.1351	DGMG (16:0)	ND	ND	✓	[44]
**46**	32.37	551.03198	-	-	96.9587/95.9509/74.9896/259.0125	Unknown	✓	ND	✓	
**47**	32.70	559.3117 [(M + FA) − H]^−^	C_27_H_465_O_9_	0.7	277.2169/253.0930	MGMG 18:3	✓	✓	✓	[14]
**48**	33.07	452.2789	C_21_H_44_O_7_NP	4.0	255.2327/196.0372/214.0482	l-PE 16:0	✓	✓	ND	[45]
**49**	33.63	291.1970	C_18_H_28_O_3_	5.0	185.1177	Oxo octadecatetraenoic acid	✓	ND	ND	[14]
**50**	38.66	263.1291	C_15_H_20_O_4_	5.0	219.1384	(±)-Abscisic acid	✓	ND	ND	
**51**	41.27	953.5477 [(M + FA) − H]^−^	C_50_H_82_O_17_	0.8	277.2169/249.1856/397.1350/101.0230	DGDG (16:3; 18:3)	✓	✓	✓	[14]
**52**	43.19	981.5782 [(M + FA) − H]^−^	C_52_H_86_O_17_	0.1	277.2168/397.1349/101.0231	DGDG (18:3; 18:3)	✓	✓	✓	[14]

Rt (min): retention time in minutes.

### 3.2. GC-qMS Analysis

According to the HS–SPME/GC–MS analysis, overall 89 VOCs belonging to ketones (9), esters (6), aldehydes (16), pyrazines (3), alcohols (21), terpenes (6), glucosinolate hydrolysis products (8), N-compounds (4), acids (10), and others (6) were identified or tentatively identified in leaves from the three different phenological stages of *E. sativa* (M, V, and R). The VOCs detected in the different rocket samples were in line with previous data [16,17,18,19,20,21,22,23], as reported in Table 2, in which the abbreviation codes, the experimental Kovats indexes, and the identification method are also listed.

The HS–SPME/GC–MS semi-quantitative data, calculated as the percentage ratio of the respective peak area to the peak area of 2-octanone (IS) (RPA%), were treated by a one-way Anova test with the MetaboAnalyst 5.0 web-based tool, to explore the effects of the three different developmental stages on the VOCs’ profiles. The data complexity was reduced, as described in the experimental section. Following the Anova, significant statistical differences in the volatile content among the M, V, and R plants were evidenced (*p* < 0.05; Supplementary Table S1).

Aldehydes were the most abundant volatiles in the samples from M and V plants, accounting for about 66% and 51% of the total VOCs, respectively, with 2-hexenal (Ald9) as the main constituent (56% and 35% of the total volatiles in M and V samples, respectively) (Table S1). On the other hand, in the R plants alcohols were the most representative metabolites (76% of the total VOCs), with *cis*-3-hexen-1-ol (Alc12) as the most abundant compound (62% of the total volatiles) (Table S1). It is noteworthy that recently, Bell et al. (2021) reported that even when observed at high concentration, Alc12 can only confer weak fresh and green notes in rocket leaves, while Ald9, if observed in relatively low content, can impart a slightly strong flavour, defined as fresh, green, leafy, and apple-like [16].

Acids and ketones constituted the third most abundant classes (about 4% of the total VOCs) in the leaves of M plants, while in both V and R plants isothiocyanates (ITCs), the enzymatic hydrolysis products of glucosinolates, were the third class of VOCs from a quantitative point of view (10 and 5% of the total volatiles, in the V and R rocket, respectively) (Table S1). ITCs, which are principally responsible for the typical pungent and Brassicaceae-like odour and flavour of fresh rocket, were described to extend potential beneficial effects on human health [14,24].

### 3.3. Multivariate Data Analysis

To identify volatile and non-volatile compounds associated with the different phenological stages of *E. sativa*, multivariate data analysis (MVA) was performed using a pseudo-targeted approach. A data matrix was constructed by manually integrating the peak areas of the compounds listed in Table 1 and Table 2. Raw UHPLC/Q-Exactive/MS data were processed using MZmine 2.38, and the resulting matrix was analysed with SIMCA^®^-P software.

Before analysis, data were log-transformed and scaled to unit variance. Principal component analysis (PCA) was initially used to explore sample clustering, while partial least squares discriminant analysis (PLS-DA) was applied for the supervised classification and identification of discriminant metabolites. The optimal number of components was selected based on R^2^X and Q^2^X values.

**Figure 5:**
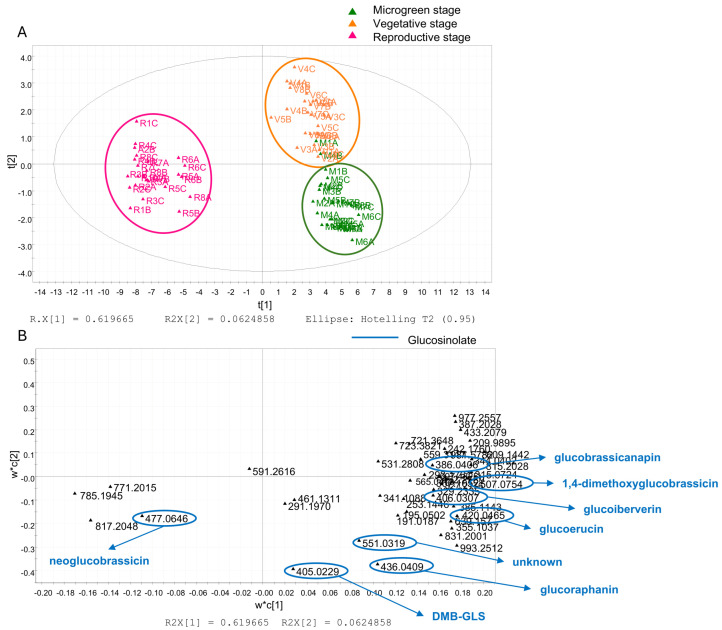
PLS-DA based on LC–HRMS pseudo-targeted analysis. The score scatter plot (**A**) is coloured to distinguish microgreen (M, green), vegetative (V, orange) e, and reproductive (R, fuchsia) stages. The loading scatter plot (**B**) highlights glucosinolates.

Figure 5 show the PLS-DA based on LC–HRMS data. The score scatter plots display the spatial distribution of the samples and reveal a clear separation according to phenological stage, with distinctive clusters.

In particular, the LC–HRMS score scatter plot (Figure 5A) shows a clear separation of the microgreen (M) and vegetative (V) stages from the reproductive (R) stage along the t[1] axis, which explains 61.97% of the total variance. This component effectively discriminates against the phenological stages, highlighting clear metabolic differences. Additionally, the separation between M and V along the t[3] axis, which accounts for 6.25% of the total variance, suggests subtle metabolic shifts occurring within the early developmental stages.

The corresponding loading plot (Figure 5B) highlights key marker compounds, revealing that several glucosinolates contributed to the observed separation. Most were predominant in the M stage, including glucoraphanin (**6**), glucoiberverin (**10**), glucoerucin (**16**), DMB-GLS (**32**), 1,4-dimethoxyglucobrassicin (**36**), and an unknown compound classified as glucosinolate (**46**). The V stage was characterised by higher levels of glucobrassicanapin (**31**), while the R stage showed a predominance of neoglucobrassicin (**27**).

**Figure 6:**
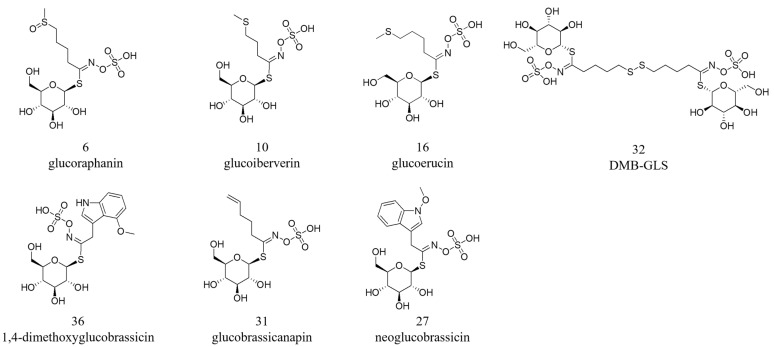
Chemical structures of glucosinolate biomarkers identified by pseudo-targeted PLS-DA in plant extracts at the microgreen, vegetative, and reproductive stages.

The chemical structures are shown in Figure 6.

**Figure 7:**
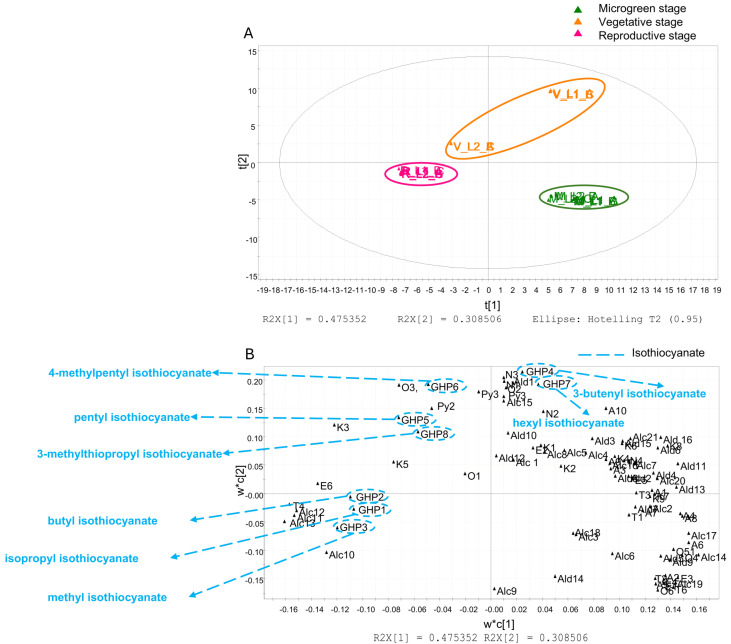
PLS-DA based on HS–SPME/GC–MS analysis. The score scatter plot (**A**) is coloured to distinguish microgreen (M, green), vegetative (V, orange) e, and reproductive (R, fuchsia) stages. The loading scatter plot (**B**) highlights isothiocyanates.

Similarly, the score scatter plot of the HS–SPME/GC–MS data (Figure 7A) shows a clear separation among the three phenological stages, indicating substantial changes in volatile profiles during plant growth. The first component accounted for 47.5% of the variance, while the second explained 30.9%. M-stage plants are separated from the others by their position in the lower right quadrant of the plot, while V-stage plants are in the upper right quadrant. Samples from R-stage plants are positioned on the left side of the plot.

**Figure 8:**
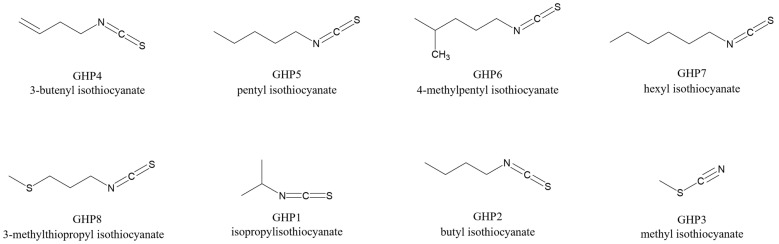
Chemical structures of isothiocyanate biomarkers identified by pseudo-targeted PLS-DA in plant extracts collected at the microgreen, vegetative, and reproductive stages.

Indeed, the corresponding loading scatter plot (Figure 7B) identified specific isothiocyanates as key discriminant metabolites, particularly abundant at the V and R stages. The chemical structures are shown in Figure 8. In detail, V stage was characterised by higher levels of 3-butenyl isothiocyanate (**GHP4**), pentyl isothiocyanate (**GHP5**), 4-methylpentyl isothiocyanate (**GHP6**), hexyl isothiocyanate (**GHP7**) and 3-methylthiopropyl ITC (**GHP8**), while the R stage was associated with increased levels of isopropyl isothiocyanate (**GHP1**), butyl isothiocyanate (**GHP2**), and methyl isothiocyanate (**GHP3**). These findings indicate that the composition and chain structure of isothiocyanates shift across phenological stages, reflecting developmental regulation of glucosinolate hydrolysis pathways.

**Figure 9:**
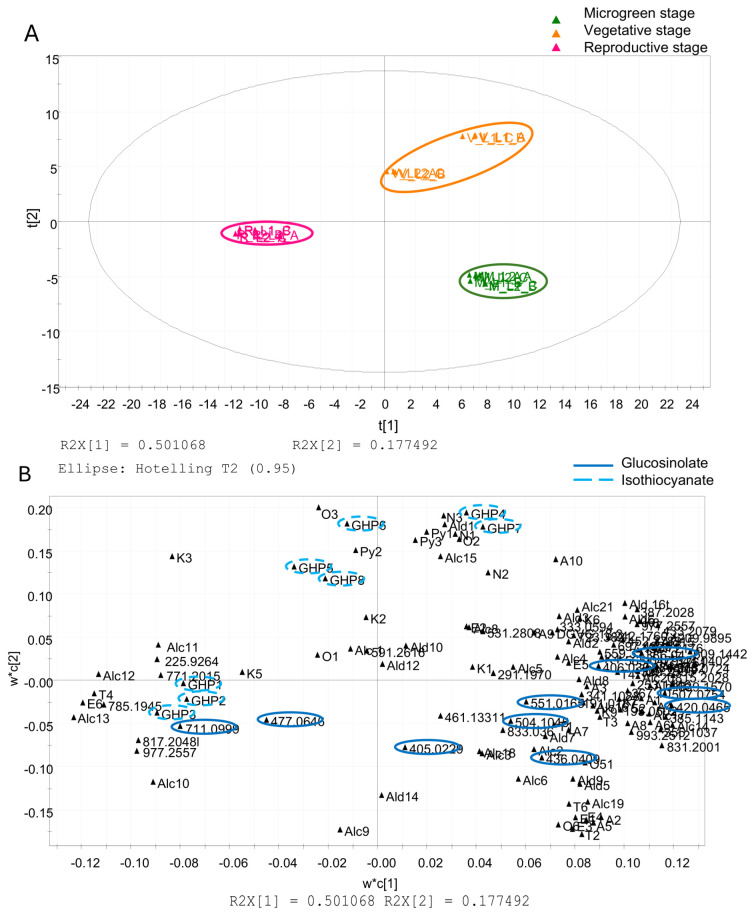
PLS-DA obtained through data fusion of LC–MS and HS–SPME/GC–MS data. The score scatter plot (**A**) is coloured to distinguish microgreen (M, green), vegetative (V, orange), and reproductive (R, fuchsia) stages. The loading scatter plot (**B**) highlights glucosinolates (plain line) and isothiocyanates (dotted line).

The PLS-DA resulting from the combination of variables corresponding to non-volatile and volatile metabolites through a data fusion approach is reported in Figure 9.

The first two components accounted for a cumulative variance of R^2^X = 0.678 (R^2^X[1] = 0.501; R^2^X[2] = 0.177), reflecting a well-defined latent structure underlying the metabolic reprogramming occurring throughout plant development.

The score scatter plot (Figure 9A) revealed distinct clustering of samples along the growth stage, while the corresponding loading scatter plot (Figure 9B) highlighted the metabolites driving group separation.

M-stage samples were positively associated with intact glucosinolates, indicating an early-stage accumulation of the biosynthetic precursors. Conversely, R-stage samples exhibited strong associations with the products of hydrolysis, including isothiocyanates, consistent with an intensification of the catabolic processes. V-stage samples displayed an intermediate profile, reflecting a transitional metabolic state characterised by the coexistence of precursor and degradation compounds.

## 4. Discussion

In this work, the secondary metabolic profile of rocket was investigated during microgreen, vegetative, and reproductive stages to highlight metabolism evolution during the plant life cycle and assess potential biological and nutritional implications. Metabolism changes were studied in plants cultivated following an innovative approach for precision plant growing, in which light regime, air flow and environmental temperature are controlled in order to minimise the metabolic variability induced by environmental factors.

The results reported in this work indicate that GSLs are key components in secondary metabolism evolution of *E. sativa*. In fact, microgreens displayed a higher content of GSLs compared to older rocket plants and also indicating an accumulation of their biosynthetic precursors in the early developmental stages of rocket. This result is consistent with recent findings that cruciferous microgreens can contain several-fold more GLSs than mature plants [46,47].

It has been reported in the scientific literature that, following plant tissue damaging and the consequent activation of the glucosinolate–myrosinase system, GSLs can release nitriles and thiocyanates, and these metabolites display antimicrobial and anti-herbivore effects, thus suggesting that GSLs are involved in plant resistance to biotic stresses [48,49].

Under this point of view, GSLs accumulation in *E. sativa* seedlings can be seen as a plant metabolism adaptation aimed to protect fleshy young tissues from soil-borne pathogens and parasites that frequently attack plants at this developmental stage [5,6].

The total GSL concentration generally declined during the plant growth—either due to a dilution in the increased biomass and/or to metabolic utilization in the growth processes—nevertheless the content of selected GSL increased again during flowering.

This finding suggests that a shift in GSL biosynthetic pathways takes place during the metabolic evolution of the plant, possibly to promote a specific plant defence in the early growth stage and more targeted defence in mature plants.

The production of secondary metabolites in *E. sativa* appears, therefore, to be dynamically regulated during the plant development to face different plant needs as they evolve from the seedling to the reproductive stage.

In our work, higher levels of the aliphatic glucobrassicanapin were detected in the plants during the vegetative stage, whereas greater accumulation of indolic neoglucobrassicin were recorded during the transition to the reproductive stage. The evolution in glucosinolate composition is also related to changes in the plant’s nutritional and antioxidant properties.

Rocket microgreens were also richer in antioxidant phytochemicals that can increase cellular defences of plantlets to the oxidative stress.

The antioxidant mechanisms that protect young *E. sativa* plants from oxidative stress may also be responsible for similar protective effects in human cells. This suggests continuity between plant defense strategies and the potential health benefits of their consumption.

It has also been demonstrated that the glucosinolate-rich fraction of *E. sativa* leaves provides significant oxidative protection due to Fe^2+^ chelation and to suppression of H_2_O_2_-induced reactive oxygen species in human cells [50].

Previous studies reported that *E. sativa* seeds and sprouts are particularly enriched in glucoerucin, which represents the predominant glucosinolate at these developmental stages, since they occur in much higher concentrations than in mature leaves.

Both glucoerucin and erucin act as hydroperoxides scavengers, generating glucoraphanin and sulforaphane. This transformation contributes to cellular protection from oxidative stress through (a) activation of phase II detoxification enzymes, (b) direct neutralization of hydrogen peroxide and organic hydroperoxides, and (c) generation of sulforaphane, a well-recognised inducer of cytoprotective enzymes.

Although erucin is not classified as a chain-breaking antioxidant, its protective potential can be strengthened when combined with other redox-active nutrients such as vitamin E or vitamin C. Therefore, the regular intake of fresh, uncooked rocket may provide an effective and natural means of enhancing antioxidant defences, highlighting the nutritional relevance of this traditionally underexploited crop [51].

This result is consistent with recent findings that cruciferous microgreens can contain several-fold more GLSs than mature plants.

The LC–HRMS approach combined with multivariate analysis of non-volatile compounds overall detected in the different growth stages of the rocket samples highlighted that GSLs appear to be key components in the differentiation of the growth stages. A higher content of GSLs is mainly observed in microgreens, indicating an early-stage accumulation of their biosynthetic precursors.

Our findings agree with the literature [52] reporting that in the Brassicaceae species sprouts and microgreens contain significantly higher GSLs amounts than mature plants, thus suggesting that microgreens can be considered valuable supplements of these compounds for a human diet. Our results also agree with those obtained comparing the GLSs content in microgreens and in other developmental stages in red cabbage (*Brassica alboglabra* and *B. rapa*) [53,54,55]. In our work, the GSLs profile of rocket was carefully characterised across microgreen, vegetative, and reproductive stages to assess potential biological implications. Specifically, glucoraphanin, glucoiberverin, glucoerucin, DMB-GLS, and 1,4-dimethoxyglucobrassicin were identified as biological markers of rocket microgreens. Among the GLSs most studied for their biological activities are glucoraphanin, glucoiberverin, and glucoerucin.

Glucoraphanin, the precursor of sulforaphane, is well documented for its chemopreventive properties, including modulation of carcinogen metabolism, induction of phase II detoxification enzymes and activation of the Nrf2/ARE pathway. It also modulates cytochrome P450 enzymes, induces apoptosis, and inhibits cancer cell proliferation and metastasis. [56]. These properties collectively contribute to reduced oxidative stress and inhibition of tumor formation [57,58,59]. Glucoiberverin displays antiproliferative and antibacterial activities, which could complement the overall bioactivity of the plant matrix [60]. Glucoerucin (hydrolysed to erucin) plays a central role in cardiovascular health. Erucin acts as a hydrogen sulfide (H_2_S) donor, promoting vasodilation, improving endothelial function and providing cytoprotective effects on vascular cells. Several studies have demonstrated that erucin can attenuate oxidative stress, reduce inflammation, and modulate vascular smooth muscle cell function, suggesting a direct cardioprotective action [61,62,63,64,65].These properties highlight its potential in maintaining cardiovascular homeostasis and mitigating risk factors associated with vascular diseases.

Collectively, the high levels of these bioactive GLSs in rocket microgreens support their classification as a potential functional food and highlight this developmental stage as a concentrated source of health-promoting compounds effective in the prevention of oxidative stress-related disorders, cardiovascular dysfunction, and inflammation-associated diseases.

The analysis of the VOCs profiles further revealed stage-specific shifts. Microgreens (M) clustered distinctly from later stages, reflecting a different volatile fingerprint. The vegetative (V) stage was characterised by higher levels of medium-chain aliphatic ITCs (e.g., 3-butenyl, pentyl, 4-methylpentyl, and hexyl ITCs), whereas the reproductive (R) stage had more short-chain ITCs (isopropyl, butyl, and methyl ITCs). These ITC patterns likely reflect the underlying GSL pool and myrosinase activity at each stage. Isothiocyanates are major contributors to rocket’s characteristic pungent aroma, and changes in chain length and composition alter the sensory profile [66].

The characterization of the whole VOCs’ profile is pivotal to evaluate the sensory trait, which is strictly related to the synergic action of all the VOCs. In this regard, although ITCs are recognised as major contributors to the typical flavour of *E. sativa*, several studies highlighted that other compounds can also contribute to the typical pungent aroma of fresh Brassicaceae plants [66,67,68]. The multiblock data fusion approach enhanced model resolution and facilitated a more comprehensive interpretation of the metabolomic shifts associated with *E. sativa* ontogeny.

## 5. Conclusions

The results obtained in this work clearly indicate that the growth stage of *E. sativa* affects the plant secondary metabolism. The chemometric approach based on data fusion carried out in the present study on plants grown under a precision agriculture approach, clearly indicated that GSLs are more abundant in microgreens, whereas ITCs tend to accumulate in mature leaves of *E. sativa*, thus suggesting a dynamic modulation of secondary metabolism during the plant life cycle in response to different plant needs related to plant development. Even though a definitive relation between metabolic profiles of GLSs/ITCs and bioactivity is yet to be identified, our results emphasise the potential of microgreens as a functional food useful in the prevention of chronic diseases or as basic ingredients for the development of nutraceutical products with health-promoting properties. Moreover, our findings provide clear indications to farmers for optimizing the nutritional and sensory qualities of rocket, when used as food. Since the content and proportion of individual glucosinolates in microgreens is affected by growing conditions (cultivation period, temperature, light, and soil nutrients, etc.), an effective control of the cultivation environment through precision agriculture techniques, as applied in this work, can support farmers in tailoring the nutritional and sensorial quality of rocket. Further investigations are needed to evaluate whether and at what extent different environmental conditions, such as light regimes, airflow, and thermoperiodic conditions, can affect the GLSs production in microgreens.

## Figures and Tables

**Table 2:** Volatile organic compounds detected in plants at microgreen (M), vegetative (V), and reproductive (R) stages and their identification codes.

Metabolite	Code	RIsp ^a^/RIt ^b^	ID ^c^	M	V	R	Metabolite	Code	RIsp ^a^/RIt ^b^	ID ^c^	M	V	R
**Ketones**													
Acetone	K1	812/816	RI/MS/S	✓	✓	✓	*trans*-2-Hexen-1-ol	Alc13	1394/1399	RI/MS/S	ND	ND	✓
2-Butanone	K2	905/910	RI/MS/S	✓	✓	✓	1-Octen-3-ol	Alc14	1446/1448	RI/MS/S	✓	✓	ND
3-Pentanone	K3	980/997	RI/MS/S	ND	✓	✓	1-Heptanol	Alc15	1460/1460	RI/MS/S	ND	✓	ND
4-Methyl-2-pentanone	K4	1010/1019	RI/MS	✓	✓	✓	2-Ethyl-1-hexanol	Alc16	1484/1488	RI/MS/S	✓	✓	✓
1-Penten-3-one	K5	1019/1021	RI/MS/S	✓	✓	✓	2,6-Dimethylcyclohexanol	Alc17	1111/1099	RI/MS/S	✓	✓	✓
2,3-Pentanedione	K6	1050/1050	RI/MS/S	✓	✓	✓	2-Furanmethanol	Alc18	1666/1661	RI/MS/S	✓	ND	ND
6-Methyl-5-hepten-2-one	K7	1348/1342	RI/MS/S	✓	✓	✓	Benzenemethanol, .alpha.-methyl	Alc19	2093/2092	RI/MS	✓	ND	ND
3,5-Octadien-2-one	K8	1524/1525	RI/MS	✓	✓	✓	Benzenmethanol	Alc20	1864/1861	RI/MS/S	✓	✓	ND
Acetophenone	K9	1660/1700	RI/MS/S	✓	✓	✓	Benzenethanol	Alc21	1880/1881	RI/MS/S	✓	✓	ND
**Esters**							**Terpenes**						
Methyl acetate	E1	839/800	RI/MS/S	✓	ND	ND	Limonene	T1	1204/1204	RI/MS/S	✓	✓	✓
Ethyl acetate	E2	863/875	RI/MS/S	✓	✓	✓	1.8-Cineol	T2	1198/1199	RI/MS/S	✓	ND	ND
2-Methyl-2-butenoate	E3	1175/1188	RI/MS	✓	ND	ND	Linalool	T3	1532/1529	RI/MS/S	✓	✓	ND
Methyl hexanoate	E4	1190/1190	RI/MS/S	✓	ND	ND	β-Cyclocitral	T4	1582/1579	RI/MS/S	✓	✓	✓
Methyl-3-hexenoate	E5	1253/1258	RI/MS/S	✓	✓	ND	Veratrol	T5	1706/1700	RI/MS	ND	ND	✓
*cis*-3-Hexenyl acetate	E6	1321/1320	RI/MS/S	ND	ND	✓	β-Ionone	T6	1909/1910	RI/MS/S	✓	ND	ND
**Aldehydes**							**Glucosinolate Hydrolysis Products (GHPs)**						
Butanal	Ald1	883/880	RI/MS/S	ND	✓	ND	Isopropyl ITC	GHP1	1177/1167	RI/MS/S	ND	ND	✓
2-Methyl butanal	Ald2	919/923	RI/MS/S	✓	✓	ND	Butyl ITC	GHP2	1308/1300	RI/MS/S	ND	ND	✓
3-Methyl butanal	Ald3	920/922	RI/MS/S	✓	✓	ND	Methyl ITC	GHP3	1228/1230	RI/MS/S	✓	✓	✓
Pentanal	Ald4	1013/1013	RI/MS/S	✓	✓	ND	3-Butenyl ITC	GHP4	1459/1448	RI/MS	ND	✓	ND
2-Butenal	Ald5	1035/1033	RI/MS	✓	✓	✓	Pentyl ITC	GHP5	2242/2250	RI/MS	✓	✓	✓
Hexanal	Ald6	1084/1082	RI/MS/S	✓	✓	✓	4-Methylpentyl ITC	GHP6	1529/1531	RI/MS	✓	✓	✓
2-Pentenal	Ald7	1140/1150	RI/MS/S	✓	✓	✓	Hexyl ITC	GHP7	1588/1600	RI/MS/S	✓	✓	✓
Heptanal	Ald8	1188/1190	RI/MS/S	✓	✓	✓	3-Methylthiopropyl ITC	GHP8	1979/1980	RI/MS/S	ND	✓	✓
2-Hexenal	Ald9	1242/1250	RI/MS/S	✓	✓	✓	**N-compounds**						
Octanal	Ald10	1286/1283	RI/MS/S	✓	✓	✓	Hexanitrile	N1	1315/1300	RI/MS/S	ND	✓	ND
2-Heptenal	Ald11	1323/1326	RI/MS/S	✓	✓	ND	5-Methyl-hexanitrile	N2	1350/1350	RI/MS	✓	✓	ND
Nonanal	Ald12	1390/1380	RI/MS/S	✓	✓	✓	Heptanitrile	N3	1405/1404	RI/MS/S	ND	✓	ND
2,4-Heptadienal	Ald13	1464/1460	RI/MS/S	✓	✓	✓	Butanenitrile, 4-(methylthio)-	N4	1812/1810	RI/MS	✓	✓	ND
Decanal	Ald14	1506/1502	RI/MS/S	✓	✓	✓	**Acids**						
Benzaldehyde	Ald15	1530/1530	RI/MS/S	✓	✓	ND	Acetic acid	A1	1445/1445	RI/MS/S	✓	✓	ND
Benzenacetaldehyde	Ald16	1616/1621	RI/MS	✓	✓	ND	2,2-Dimethylpropanoic acid	A2	1227/1225	RI/MS	✓	ND	ND
**Pyrazines**							Butanoic acid	A3	1637/1635	RI/MS/S	✓	✓	ND
2-Methoxy-3-(1-methylethyl)pyrazine	Py1	1509/1500	RI/MS	✓	✓	✓	2-Methylbutanoic acid	A4	1682/1680	RI/MS/S	✓	✓	ND
2-Methoxy-3-(1-methylpropyl)pyrazine	Py2	1514/1520	RI/MS/S	✓	✓	✓	Pentanoic acid	A5	1733/1729	RI/MS/S	✓	ND	ND
2-Methoxy-3-(2-methylpropyl)pyrazine	Py3	1492/1151	RI/MS	ND	✓	✓	Hexanoic acid	A6	1813/1813	RI/MS/S	✓	✓	✓
**Alcohols**							3-Hexenoic acid	A7	1942/1940	RI/MS/S	✓	✓	✓
2-Propanol	Alc1	975/974	RI/MS/S	✓	✓	✓	2-Hexenoic acid	A8	1042/1043	RI/MS/S	✓	✓	ND
Ethanol	Alc2	955/945	RI/MS/S	✓	✓	✓	Octanoic acid	A9	2055/2050	RI/MS/S	✓	✓	ND
2-Methyl-1-propanol	Alc3	1097/1095	RI/MS/S	✓	ND	ND	Nonanoic acid	A10	2174/2178	RI/MS/S	✓	✓	ND
1-Butanol	Alc4	1125/1125	RI/MS/S	✓	✓	✓	**Others**						
1-Penten-3-ol	Alc5	1188/1189	RI/MS/S	✓	✓	✓	2-Ethylfuran	O1	950/947	RI/MS/S	✓	✓	✓
Isoamyl alcohol	Alc6	1222/1226	RI/MS/S	✓	ND	ND	Toluene	O2	1035/1035	RI/MS/S	ND	✓	ND
1-Pentanol	Alc7	1260/1271	RI/MS/S	✓	✓	✓	Tetrahydrothiophene	O3	1077/1073	RI/MS	ND	✓	✓
*trans*-2-Penten-1-ol	Alc8	1335/1341	RI/MS/S	✓	✓	✓	2-Butoxyethanol	O4	1405/1411	RI/MS	✓	✓	ND
*cis*-2-Penten-1-ol	Alc9	1272/1270	RI/MS/S	✓	✓	✓	1,3-Di-tert-butylbenzene	O5	1426/1428	RI/MS/S	✓	✓	✓
1-Hexanol	Alc10	1340/1341	RI/MS/S	✓	✓	✓	5-Ethyl-2(5H)-furanone	O6	1910/1907	RI/MS	✓	ND	✓
*trans*-3-Hexen-1-ol	Alc11	1386/1386	RI/MS/S	✓	✓	✓							
*cis*-3-Hexen-1-ol	Alc12	1390/1389	RI/MS/S	✓	✓	✓							

^a^ RIsp, relative retention indexes calculated versus n-alkanes (C_8_–C_20_) on the HP-INNOWax column; ^b^ RIt teorical relative retention indexes reported in literature, ^c^ identification method as indicated by the following: RI, Kovats retention index on an HP-INNOWax column; MS, NIST, and Wiley libraries spectra; S, co-injection with authentic standard compounds on the HP-INNOWax column.

## Data Availability

The data used to support the findings of this study can be made available by the corresponding author upon request.

## Supplementary Materials

The following supporting information can be downloaded at: https://www.mdpi.com/article/10.3390/foods14234148/s1, Table S1: VOCs identified by one-way ANOVA and post hoc analysis, found as statistically significant for *p* < 0.05 M vs. V vs. R.
